# Recognition of the genus *Thaumatophyllum* Schott − formerly Philodendron subg. Meconostigma (Araceae) − based on molecular and morphological evidence

**DOI:** 10.3897/phytokeys.98.25044

**Published:** 2018-05-02

**Authors:** Cassia Mônica Sakuragui, Luana Silva Braucks Calazans, Leticia Loss de Oliveira, Érica Barroso de Morais, Ana Maria Benko-Iseppon, Santelmo Vasconcelos, Carlos Eduardo Guerra Schrago, Simon Joseph Mayo

**Affiliations:** 1 Universidade Federal do Rio de Janeiro, Centro de Ciências da Saúde, Instituto de Biologia, Departamento de Botânica, Av. Carlos Chagas Filho, 373 – Sala A1-088 – Bloco A, Ilha do Fundão, Rio de Janeiro, RJ, CEP 21941-902, Brazil; 2 Universidade do Estado do Rio de Janeiro, Centro de Educação e Humanidades, Instituto de Aplicação Fernando Rodrigues da Silveira, Rua Santa Alexandrina, 288, Rio Comprido, Rio de Janeiro, RJ, CEP 20261-232, Brazil; 3 University of Zurich, Department of Systematic and Evolutionary Botany, 8008 Zurich, Switzerland; 4 Universidade Federal de Pernambuco, Centro de Biociências/Genética, Av. Prof. Moraes Rego, 1235, Recife, PE, CEP 50670-420, Brazil; 5 Instituto Tecnológico Vale, Rua Boaventura da Silva, 955, Nazaré, Belém, PA, CEP 66055-090, Brazil; 6 Universidade Federal do Rio de Janeiro, CCS, Instituto de Biologia, Departamento de Genética, Av. Carlos Chagas Filho, 373 – Sala A2-092 – Bloco A, Ilha do Fundão, Rio de Janeiro, RJ, CEP 21941-902, Brazil; 7 Royal Botanic Gardens, Kew, Richmond, Surrey, TW9 3AB, UK

**Keywords:** chromosomes, molecular phylogeny, morphology, nomenclature, *Philodendron*, *Thaumatophyllum*

## Abstract

Philodendron subgenus Meconostigma has been a well-circumscribed group since 1829. Members of this group are easily distinguished by diagnostic morphological characters as well as by a distinct ecology and geographical distribution. Based on molecular, morphological and cytological evidence, we propose the recognition of P. subg. Meconostigma as a distinct genus, *Thaumatophyllum* Schott. We also present the necessary new combinations, an emended key and some nomenclatural and taxonomic corrections regarding 21 names of *Thaumatophyllum*.

## Introduction

*Philodendron* Schott is the second most species-rich and diverse genus in the family Araceae and also in the “*Homalomena* clade” (sensu Cusimano et al. 2011), comprising 487 formally recognised species (Boyce and Croat 2018). The genus ranges from northern Mexico to southern Uruguay (Mayo et al. 1997), most commonly in tropical humid forests as epiphytes and hemi-epiphytes. Most rarely, it also occurs as terrestrial plants in open habitats (e.g. seasonal dry forests of South America).

Cabrera et al. (2008) published a family-wide molecular phylogeny that included species from 102 genera. Cusimano et al. (2011) re-analysed and augmented a molecular data set with a more complete genus sampling and compared the resulting phylogeny with morphological and anatomical data, proposing informal names for the suprageneric clades. The “*Homalomena* clade” (composed of the genera *Adelonema* Schott, *Cercestis* Schott, *Culcasia* P.Beauv., *Furtadoa* M.Hotta, *Homalomena* Schott and *Philodendron* Schott) was recovered in both molecular and morphological analyses and was supported by the occurrence of sclerotic hypodermis and resin canals in the roots and absence of endothecial thickenings in the anthers (present in *Homalomena*). The clade is composed of two sister groups: “Culcasieae clade” (*Cercestis, Culcasia*) and “*Philodendron* clade” (*Furtadoa*, *Homalomena*, *Philodendron*). Mayo et al. (2013) gave an alphabetical table of the clades that is a useful complement to the listing in Cusimano et al. (2011).

**Figure 1.**
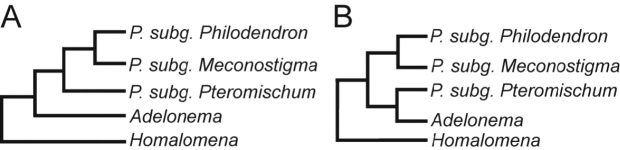
*Philodendron*, *Homalomena* and *Adelonema* phylogenetic relationships markers by previous publications. **A** Gauthier et al. (2008), maximum parsimony. **B** Gauthier et al. (2008), Bayesian analysis; Wong et al. (2013), Wong et al. (2016).

The evolutionary history of the “*Philodendron* clade” has been discussed in several recent papers (Tam et al. 2004, Gauthier et al. 2008, Mayo et al. 2013, Wong et al. 2013, Loss-Oliveira et al. 2014, Wong et al. 2016, Loss-Oliveira et al. 2016), as well as the relationship amongst the three subgenera of *Philodendron* as independent lineages (Gauthier et al. 2008, Loss-Oliveira et al. 2014, Loss-Oliveira et al. 2016). A question recently answered was how the Asian-Neotropical distribution of the genus *Homalomena* originates (*sensu* Mayo et al. 1997). Based on molecular evidence (Gauthier et al. 2008, Wong et al. 2013, Wong et al. 2016), the American species of *Homalomena* were recognised as a separate lineage and consequently Schott’s old genus *Adelonema* was recognised once more (Wong et al. 2016). The “*Philodendron* clade”, still needs better phylogenetic resolution for two other lineages: *Homalomena* + *Furtadoa* and *Philodendron* subgenera *Philodendron* and *Pteromischum*. Several research articles (Wong et al. 2016, Loss-Oliveira et al. 2016) have proposed different hypotheses for the relationship amongst these lineages as summarised in Fig. 1.

The recent recognition of the genus *Adelonema* for the American species of *Homalomena* (Wong et al. 2016) makes the genus *Philodendron* paraphyletic in some of the current proposed phylogenetic hypothesis (Figs 1B, 3A). The most recent studies (Loss-Oliveira et al. 2016, Vasconcelos 2015) recovered two major lineages: P. subg. Meconostigma (= *Thaumatophyllum*) and Philodendron subg. Philodendron plus subg. Pteromischum. Vasconcelos (2015) recovered P. subgenus Pteromischum as monophyletic and sister clade to P. subg. Philodendron.

Philodendron subgenus Meconostigma (= *Thaumatophyllum*) has been a well-circumscribed group since the genus *Philodendron* was first recognised taxonomically by Schott (1829). It is now comparatively well-studied taxonomically; the last taxonomic revision included 15 species (Mayo 1991, with updates by Gonçalves and Salviani 2002, who recognised 19 species). Members of this subgenus are easily distinguished by diagnostic morphological characters as well as by a distinct ecology and geographical distribution that ranges from the Amazonian and Atlantic forests to the savannah-like landscapes of the Cerrado biome (Mayo 1991).

Based on the evidence now accumulated (most recently, by Calazans et al. 2014, Loss-Oliveira et al. 2014, 2016), we propose the recognition of *P.* subg. *Meconostigma* as a distinct genus, *Thaumatophyllum* Schott (1859), a taxon that was accepted by experts as recently as Bunting (1980). Barroso (1962) was the first botanist to formally assign the name *Thaumatophyllum* to the synonymy of *Philodendron* and Mayo and Barroso (1979) gave a detailed account of the confusion that had misled previous authors regarding the floral morphology of *T. spruceanum*. The aim of this paper is, therefore, to formally propose this change of status and validly publish the necessary new combinations. We also provide an emended key and some nomenclatural and taxonomic corrections concerning six names in this genus.

## Methods

### Taxon and gene sampling

We have sampled data for 110 extant species of *Philodendron*, 21 species of *Thaumatophyllum* and six species of *Homalomena* and five of *Adelonema* of the nuclear 18S and external transcribed spacer (ETS) and the chloroplast *trn*K intron, maturase K (*matK*) genes, *trn*L intron, *trn*L-*trn*F intergenic spacer. Species from the genera *Cercestis*, *Culcasia*, *Colocasia*, *Dieffenbachia*, *Heteropsis*, *Montrichardia*, *Nephthytis*, *Furtadoa* and *Urospatha* were included as the outgroup. The species list, the voucher and GenBank accession numbers are listed in Suppl. material 1: table 1. The majority of the used sequences were generated by a previous study of our group (Loss-Oliveira et al. 2016).

Additionally, we generated a subsampled dataset comprised of species from our original data with available ETS and 18S sequences and at least two available chloroplast sequences. This strategy aimed to reduce the impact of missing data in the concatenated analysis. This taxon sampling is described in Suppl. material 1: table 1.

### Alignment and phylogenetic analysis

The methodological approach of Loss-Oliveira et al. (2016) was followed in order to estimate individual gene trees and a supertree. We have used MAFFT 7 (Katoh and Standley 2013) to individually align the molecular markers and SeaView 4 (Gouy et al. 2010) to manually adjust them. Bayesian analysis was conducted in MrBayes 3.2.2 (Huelsenbeck and Ronquist 2001, Ronquist and Huelsenbeck 2003) for individual gene trees (Fig. 1, Suppl. material 1) using the GTR + G substitution model. The Markov chain Monte Carlo (MCMC) algorithm was run twice for 10,000,000 generations with four chains, which were sampled every 100^th^ cycle. We have applied a burn-in of 20%. Individual gene trees were used to estimate a supertree with PhySIC_IST algorithm (http://www.atgc-montpellier.fr/physic_ist/) in order to avoid the impact of missing data in the estimation (Scornavacca et al. 2008).

We have also performed phylogenetic analysis for concatenated chloroplast markers separated from nuclear markers from the subsample consisting of species with available ETS and 18S sequences and at least two chloroplast markers in order to compare the estimated trees. Both chloroplast and nuclear datasets were used to estimate trees from Maximum Likelihood and Bayesian analysis approaches.

A maximum likelihood approach was performed in PhyML, implemented in Seaview (Gouy et al. 2010). The GTR+G model of sequence evolution was used for both chloroplast and nuclear concatenated sequences.

The Bayesian analysis were performed in MrBayes 3.2.2 (Huelsenbeck and Ronquist 2001, Ronquist and Huelsenbeck 2003) using the GTR + G substitution model for both chloroplast and nuclear concatenated sequences. The MCMC algorithm was run twice for 10,000,000 generations, using four chains. Chains were sampled every 100th cycle and a burn-in of 20% was applied.

## Results

### Phylogenetic analysis

**Figure 2.**
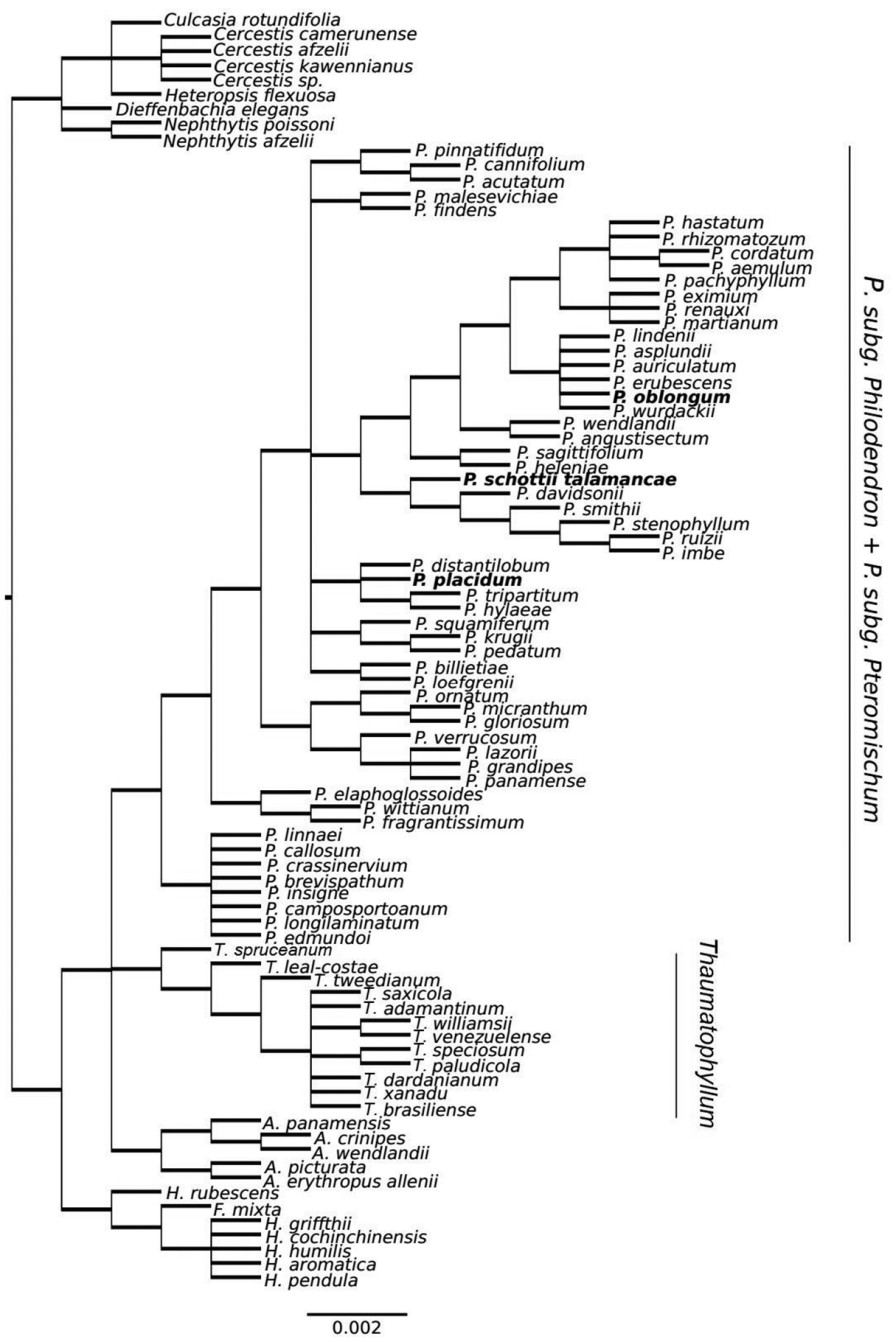
Supertree of *Philodendron*, *Thaumatophyllum*, *Adelonema* and *Homalomena* species. Names in bold are species of P. subg. Pteromischum.

As observed in Figure 2, Philodendron subg. Meconostigma was recovered as monophyletic and as a sister group of P. subg. Philodendron and P. subg. Pteromischum.

**Figure 3.**
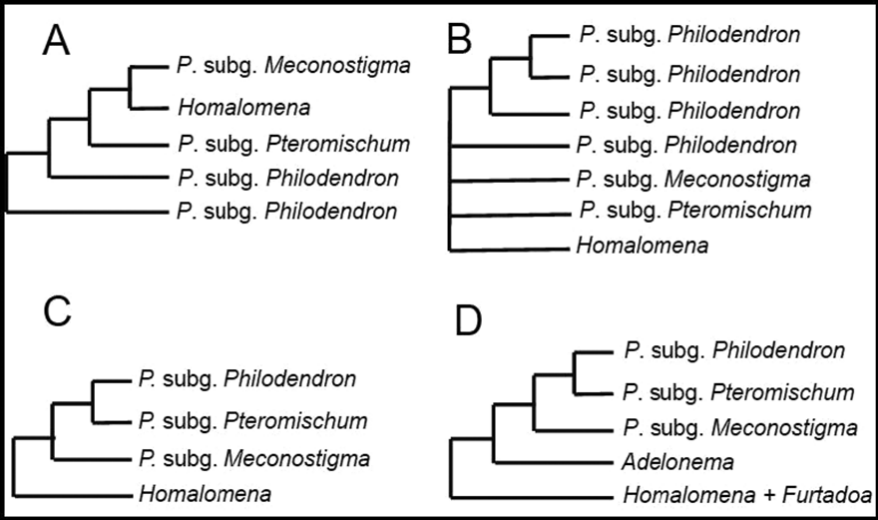
Phylogenetic relationships amongst *Philodendron*, *Thaumatophyllum*, *Homalomena* and *Adelonema* recovered by previous authors. **A** Barabé et al. (2002) **B** Tam et al. (2004) **C** Loss-Oliveira et al. (2014) **D** Vasconcelos (2015).

The subsampled chloroplast analyses (Figure 2, Suppl. material 1) were inconclusive. They presented very low posterior probabilities for Bayesian analysis (Figure 2A, Suppl. material 1), as well as very low aLRT values for Maximum Likelihood estimates (Figure 2B, Suppl. material 1). On the other hand, the results from ETS and 18S analysis (Figure 3, Suppl. material) presented very similar results for both Bayesian analysis (Figure 3A, Suppl. material 1) and Maximum Likelihood estimates (Figure 3B, Suppl. material 1), with high posterior probabilities and aLRT support, respectively. The estimated phylogenetic relationships are also very similar to those found in the estimated supertree.

## Discussion

### The genus *Thaumatophyllum* Schott

***Molecular evidence.*** Barabé et al. (2002) used the *trn*L intron and *trn*L-*trn*F intergenic region to estimate the phylogenetic relationships of 33 genera of Araceae; they included six species of *Philodendron* and found that three species of subg. Philodendron formed a sister group to a clade composed of species of *Thaumatophyllum*, subg. Pteromischum and *Homalomena*; *P. insigne* (subg. Philodendron sect. Baursia) was sister group to all these. Tam et al. (2004) analysed the *trn*L-*trn*F with the same six species of *Philodendron* within a larger analysis focused on subfam. *Monsteroideae*, but this part of their tree was largely unresolved. Gauthier et al. (2008) carried out a more complete analysis of *Philodendron* based on over 80 species using ETS and ITS markers. Their ETS tree recovered P. subg. Meconostigma as monophyletic and sister group to P. subg. Philodendron, with P. subg. Pteromischum as the basal component of *Philodendron* (Fig. 3A). In their ITS tree, the three subgenera formed a trichotomy.

Similarly, Loss-Oliveira et al. (2014) recovered P. subg. Meconostigma (= *Thaumatophyllum*) as monophyletic with 100% aLRT support and 100% posterior probability through the analysis of nuclear ETS and 18S markers and chloroplast *mat*K, *trn*K, *trn*L intron and *trn*L-*trn*F intergenic region (Fig. 3C). *Thaumatophyllum* was recovered as sister group of P. subg. Philodendron and P. subg. Pteromischum.

The analysis conducted by Vasconcelos (2015), using the chloroplast markers *rpl*32-*trn*L, *trn*V-*ndh*C and *trn*Q-5’-*rps*16 and the nuclear ITS, corroborate the monophyly of *Thaumatophyllum* and its position as sister group of P. subg. Philodendron and P. subg. Pteromischum (Fig. 3D).

Wong et al. (2016) used nuclear ITS and plastid *mat*K markers in an analysis which included Asian *Homalomena*, *Adelonema* (previously American *Homalomena*) and *Philodendron* (*sensu lato*) and also found that *Thaumatophyllum* and P. subg. Philodendron were sister groups. In this study, P. subg. Pteromischum emerged as sister to *Adelonema*.

These results are consistent with our findings that *Thaumatophyllum* is a monophyletic and isolated lineage in *Philodendron*, the sister group of P. subg. Pteromischum and P. subg. Philodendron.

***Morphological evidence.*** As here defined, *Thaumatophyllum* is a Neotropical genus composed of 21 species. It is defined by an arborescent habit, very much thickened spathe, well developed sterile intermediate zone in the inflorescence equal or longer than the staminate zone, the gynoecium always having stylar lobes and an axial vascular system independent of the funicle supply (Mayo 1991, Calazans et al. 2014). Other distinctive features of *Thaumatophyllum* are (Mayo 1991, Calazans et al. 2014): 1. sympodial articles diphyllous, internode between prophyll and preceding foliage leaf never developed, internode between prophyll and succeeding foliage leaf sometimes developed but usually very short; 2. leaf blade cordiform, sagittate or hastate, never unlobed at the base; 3. adaxial spathe resin canals J-shaped in longitudinal section, extending to the epidermal surface and secreting resin at anthesis; 4. abaxial spathe resin canals distributed throughout the abaxial parenchyma zone; 5. stamens normally long, slender, more than 3 times longer than wide (except *T. leal-costae*); 6. stamen vascular trace unbranched (French 1986); 7. style lobes always present; 8. central style dome often present; 9. separate stylar canals occasionally absent; 10. vascular plexus normally present in style; 11. basal vascular complex of gynoecium multi-stranded; 12. lobed central vascular cylinder in the roots (V.T. Rosa, personal comm.); 13. lack of cell wall thickening in the inner root endodermis and neighbouring cortical cells; and 14. collenchyma rather than sclerenchyma sheaths around root resin canals.

***Shoot morphology and arborescent habit.*** Stem architecture in *Thaumatophyllum* is similar to *Philodendron*, since the mature stems of both genera are sympodia composed of diphyllous articles (terminology after Ray 1987). However, in those species of *Thaumatophyllum* which have appreciably elongated internodes, the pattern of elongation is different from that of *Philodendron*. The position of the ‘intravaginal squamules’ (Dahlgren and Clifford 1982, Mayo 1991) is also different in the two genera and is evidence of the two contrasting patterns of internode elongation. The squamules are always found immediately above the prophyll scar in mature internodes of *Philodendron*. However, in *Thaumatophyllum*, the squamules occur immediately below the prophyll scar and often surround the foliage leaf scar as well. Also in *Thaumatophyllum* the squamules frequently persist on the adult stem and are normally spinose or aculeate projections; their number, size, shape and persistence are taxonomically useful.

***Inflorescence.*** *Thaumatophyllum* is characterised by normally solitary inflorescences in each floral sympodium and very thick, weakly constricted or unconstricted spathes with a uniformly white inner surface. In the spadix, the long staminodial zone that equals or exceeds the fertile male zone is the most useful diagnostic character and distinguishes it from the genus *Philodendron*. This long staminodial zone plays an important role in the floral biology, serving as a food resource and as the main source of the very large temperature elevations observed during flowering (Gibernau et al. 1999, Gibernau and Barabé 2000, Barabé et al. 2002, Gibernau et al. 2005).

***Pistillate flowers and the Gynoecium.*** Unlike *Philodendron*, the style lobes are conspicuous in *Thaumatophyllum* and, together, constitute the style crown (Mayo 1991); they resemble stigma lobes as they are frequently covered by stigmatic tissue but are distinct from other kinds of lobed stigma because the lobing is caused by the style apex tissues rather than differential growth of the stigma trichomes. In many species the central region of the style apex is elongated into a more-or-less cylindrical axial portion, the central dome. The central dome may be excavated itself into a pit or even a long canal and may itself have lobed margins. The gynoecial type, typical of *Thaumatophyllum*, was designated by Mayo (1986, 1989, 1991) as type A, based on a sample of only four species. Calazans et al. (2014) studied 19 out of 21 species and recognised a further three subtypes within Mayo’s type A: subtype A1: stylar body absent and stylar canals short, central stylar dome absent and compitum deep (*T. adamantinum*, *T. dardanianum*, *T. speciosum* and *T. williamsii*); subtype A2: undeveloped stylar body present with long stylar canals, central stylar dome absent and compitum shallow (*T. corcovadense*, *T. lundii*, *T. paludicola*, *T. saxicola*, *T. stenolobum*, *T. tweedieanum* and *T. uliginosum*); subtype A3: well developed stylar body present with stylar canals long, central stylar dome present and compitum shallow (*T. bipinnatifidum*, *T. brasiliense*, *T. mello-barretoanum*, *T. petraeum*, *T. spruceanum*, *T. solimoesense*, *T. undulatum* and *T. venezuelense*).

Based on molecular evidence, Loss-Oliveira et al. (2014) suggested that the common ancestor of *Thaumatophyllum* probably possessed short stylar lobes, long stylar canals, a stylar body, a vascular plexus in the gynoecium and druses in the stylar parenchyma. These authors also proposed that the morphological diversity observed in the gynoecium of *Thaumatophyllum* species is the result of an ongoing process of fusion of its floral structures and that the resulting reduction of energy wastage and increase in stigmatic surface are likely to be evolving under positive selection.

***Chromosome numbers.*** Available chromosome numbers for *Philodendron* range from 2n = 28 to 40 (Correia-da-Silva et al. 2014) with a prevalence of 2n = 32, whereas for *Thaumatophyllum* they range from 2n = 28 to 36, with a clear prevalence of 2n = 36, indicating a distinct cytological trend (Correia-da-Silva et al. 2014, Vasconcelos et al. 2017).

***Evolutionary history.*** Mayo (1988) hypothesised that *Thaumatophyllum* was the first lineage to emerge as a distinct clade from ancestral *Philodendron* and the Eastern and Southern South America species would present a higher number of plesiomorphic gynoecial characters (low number of locules and simple style) and the Amazonian species would have more apomorphic characters (high number of locules and elaborated style). Results from the morphology-based phylogenetic reconstruction of Calazans et al. (2014) partly agreed with Mayo’s (1988) findings, recognising it as a natural group and suggesting its origin and diversification within open areas of the Cerrado biome. Loss-Oliveira et al. (2016) however, based on molecular evidence, proposed that the last common ancestor of *Philodendron* occurred in Amazonia about 8.6 Ma (11.1–6.8 Ma) during the Middle/Late Miocene, and that *Philodendron* lineages occurred exclusively in Amazonia for ca. 5.0–6.0 Ma. This implies that *Thaumatophyllum*, as well as the Atlantic forest lineages, must have diverged from Amazonian ancestors. The majority of *Thaumatophyllum* species from the Cerrado would then have evolved from Atlantic forest ancestors, from the Late Miocene to the Pliocene.

***Ecology.*** *Thaumatophyllum* species have a preference for open environments with higher light intensity. The life forms vary from terrestrial to hemi-epiphytic, but can be rupicolous (*T. saxicola* and *T. adamantinum*), aquatic or subaquatic in freshwater swamps at lowland sites (*T. tweedieanum*, *T. undulatum*, *T. uliginosum*). More frequent are forest hemi-epiphytes which grow equally well in rupicolous habitats or even in open coastal sites on sand in the case of the *T. williamsii*, *T. corcovadense*, *T. bipinnatifidum* and *T. speciosum*. All the extant species have a notable preference for open habitats and the ability to tolerate a certain degree of drought.

### Taxonomic treatment

#### Thaumatophyllum

Taxon classificationPlantaeAlismatalesAraceae

Schott, Bonplandia 7: 31. 1859.

##### Type.

*Thaumatophyllum spruceanum* Schott, Bonplandia 7: 31. 1859.

##### Etymology.

from Ancient Greek “θαυματ-” (“*thaumato*-”, wonder, miracle) + “φύλλον” (“*phyllum*”, leaf); wonderful leaf, referring to the beautiful and peculiar leaves of the type species.

#### Thaumatophyllum adamantinum

Taxon classificationPlantaeAlismatalesAraceae

(Schott) Sakur., Calazans & Mayo comb. nov.

urn:lsid:ipni.org:names:77178483-1

Philodendron adamantinum Schott, Syn. Aroid. 114. 1856.

##### Type.

Brazil, Minas Gerais, Tejuco, Serro Frio, *Martius 1208* (holotype: M).

#### Thaumatophyllum bipinnatifidum

Taxon classificationPlantaeAlismatalesAraceae

(Schott ex Endl.) Sakur., Calazans & Mayo comb. nov.

urn:lsid:ipni.org:names:77178497-1

Philodendron bipinnatifidum Schott ex Endl., Gen. Pl. 1(3): 237. 1837.
Type. Illustration in Schott Icones Aroideae Nº 2640 (lectotype, designated by Sakuragui et al. 2011); Brazil, Rio de Janeiro, Arraial do Cabo, 13 Feb. 2012, *L.S.B. Calazans et al. 170* (epitype, designated by Sakuragui et al. 2011: RB).
Philodendron selloum C.Koch, Index Seminum (B) 1853 (App.): 14. 1853.
Type. Plant cultivated at Berlin Botanic Garden, *C. Koch s.n.* (lectotype, designated by Sakuragui et al. 2011: K, tracing).
Philodendron pygmaeum Chodat & Vischer, Bull. Soc. Bot. Genève 11: 299. 1919 publ. 1920.
Type. Paraguay, Paraguari, ‘Cerro Akahay’, 1914, *R.H. Chodat & W. Vischer 358* (holotype: G).

#### Thaumatophyllum brasiliense

Taxon classificationPlantaeAlismatalesAraceae

(Engl.) Sakur., Calazans & Mayo comb. nov.

urn:lsid:ipni.org:names:77178484-1

Philodendron brasiliense Engl., Fl. Bras. 3(2): 168. 1878.
Type. Brazil, Minas Gerais, Caldas, Rio Verde, Feb-Mar. 1868, *S.E. Henschen in Herb. Regnell III. Nº 1292* (lectotype, designated by Sakuragui et al. 2011: S).
Philodendron cymbispathum Engl., Bot. Jahrb. 26: 555. 1899.
Type. Brazil, Minas Gerais, *A.F.M. Glaziou 16497* (**lectotype, designated here**: B; isolectotypes: C, LE, P).

#### Thaumatophyllum corcovadense

Taxon classificationPlantaeAlismatalesAraceae

(Kunth) Sakur., Calazans & Mayo comb. nov.

urn:lsid:ipni.org:names:77178485-1

Philodendron corcovadense Kunth, Enum. Pl. 3: 49. 1841.
Type. illustration in Vellozo, Fl. Flum. 9: tab. 115. 1831 (lectotype, designated by Sakuragui et al. 2011); Brazil, Rio de Janeiro, Mangaratiba, Ilha da Marambaia, 19 Out. 2004, *M.A. Nadruz Coelho 1590* (epitype, designated by Sakuragui et al. 2011: RB).
Philodendron melanorrhizum Reitz, Sellowia 9:50, t.10. 1958.
Type. Brazil, Santa Catarina, Itajaí, Luís Alves, Braço Joaquim, 14 Oct. 1954, *R. Klein 917* (holotype: HBR; isotypes: NY, UC, US).

#### Thaumatophyllum dardanianum

Taxon classificationPlantaeAlismatalesAraceae

(Mayo) Sakur., Calazans & Mayo comb. nov.

urn:lsid:ipni.org:names:77178498-1

Philodendron dardanianum Mayo, Kew Bull. 46: 648. 1991.
Type. Brazil, Bahia, Chapadão Oriental da Bahia, 37km N from Correntina from road to Inhaúmas, 29 Apr. 1980, *Harley et al. 21963* (holotype: CEPEC; isotypes: K, MO, US).

#### Thaumatophyllum leal-costae

Taxon classificationPlantaeAlismatalesAraceae

(Mayo & G.M. Barroso) Sakur., Calazans & Mayo comb. nov.

urn:lsid:ipni.org:names:77178499-1

Philodendron leal-costae Mayo & G.M. Barroso, Aroideana 2: 82. 1979.
Type. Brazil, Bahia, Serra do Jatobá, Nossa Senhora dos Milagres, Morro do Couro, 06 Mar. 1977, *Harley et al. 19428* (holotype: CEPEC; isotypes: K, M, MO, NY, P, RB, SEL, US).

#### Thaumatophyllum lundii

Taxon classificationPlantaeAlismatalesAraceae

(Warm.) Sakur., Calazans & Mayo comb. nov.

urn:lsid:ipni.org:names:77178486-1

Philodendron lundii Warm., Vidensk. Meddel. Naturhist. Foren. Kjøbenhavn 1867: 128. 1867.
Type. Brazil, Minas Gerais, Lagoa Santa, *Warming s.n.* (holotype: C).

#### Thaumatophyllum mello-barretoanum

Taxon classificationPlantaeAlismatalesAraceae

(Burle-Marx ex G.M. Barroso) Sakur., Calazans & Mayo comb. nov.

urn:lsid:ipni.org:names:77178496-1

Philodendron mello-barretoanum Burle-Marx ex G.M. Barroso, Arch. Jard. Bot. Rio de Janeiro 15: 94. 1957.
Type. Brazil, Goiás, cultivated at Jardim Botânico do Rio de Janeiro, *Burle-Marx s.n.* (holotype: RB 97081).

#### Thaumatophyllum paludicola

Taxon classificationPlantaeAlismatalesAraceae

(E.G. Gonç. & Salviani) Sakur., Calazans & Mayo comb. nov.

urn:lsid:ipni.org:names:77178487-1

Philodendron paludicola E.G. Gonç. & Salviani, Aroideana 25: 2. 2002 publ. 2003.
Type. Brazil, Espírito Santo, São Mateus, access to Barra Nova, 21 Dec. 2000, *E.R. Salviani & L. Bernacci 1869* (holotype: UB; isotype: K).

#### Thaumatophyllum petraeum

Taxon classificationPlantaeAlismatalesAraceae

(Chodat & Vischer) Sakur., Calazans & Mayo comb. nov.

urn:lsid:ipni.org:names:77178488-1

Fig. 4.

Philodendron petraeum Chodat & Vischer, Bull. Soc. Bot. Genève 11: 296. 1919 publ. 1920.
Type. Paraguay, Tobaty between Tobaty and Barrero Grande, *R.H. Chodat & W. Vischer 349* (holotype: G).

Philodendron petraeum var. triangulare Chodat & Vischer, Bull. Soc. Bot. Genève 11: 299. 1919 publ. 1920.
Type. Paraguay, Tobaty between Tobaty and Barrero Grande, *R.H. Chodat & W. Vischer 347* (holotype: G).
Philodendron petraeum var. valenzuelae Chodat & Vischer, Bull. Soc. Bot. Genève 11: 299. 1919 publ. 1920.
Type. Paraguay, prope Valenzuela, *R. H. Chodat & W. Vischer 357* (holotype: G).

##### Remarks.

The species was previously synonymised under *P. tweedieanum* (= *T. tweedieanum*) by Croat and Mount (1988). We propose its reinstatement as an accepted species based on the following morphological differences: herbs erect and rupiculous (x herbs decumbent or rhizomatous subterranean acaulous in *T. tweedieanum*), prophyll deciduous when still herbaceous (x marcescent and persistent-membranous in *T. tweedieanum*), denudation of posterior division absent (x present in *T. tweedieanum*), presence of stylar central dome in pistillate flowers (x absence of stylar central dome in *T. tweedieanum*). Besides having a different gynoecium type (Calazans et al. 2014), the majority-rule consensus tree based on morphological characters support these species as different lineages. Furthermore, the phylogeny based on molecular characters supports the two species as separate taxa (Loss-Oliveira et al. 2014). *Thaumatophyllum petraeum* was first described for Paraguay with four varieties and are still recorded only from this country. We have no evidence to recognise the varieties as distinct taxa, except for. P. petraeum var. tobatiense Chodat & Vischer, which is a synonymous of *P. undulatum* (= *T. undulatum*).

**Figure 4.**
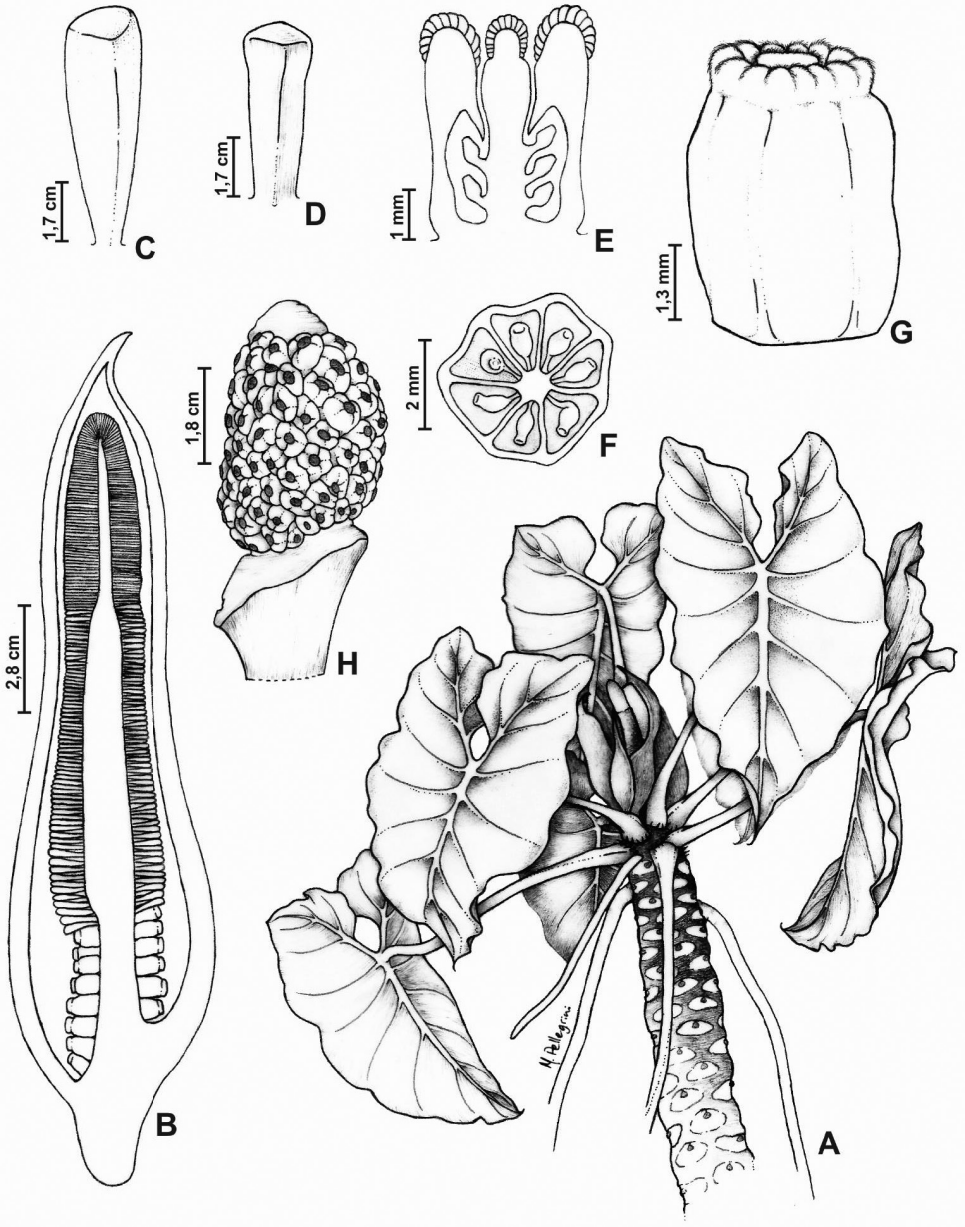
*Thaumathophyllum petreum*. **A** Habit **B** Longitudinal cut of the inflorescence **C** Staminode **D** Stamen **E** Longitudinal cut of a female flower **F** Transversal cut of a female flower showing the 6-locular ovary **G** Side view of a female flower **H** Infructescence. All from *Calazans & Morais 28* (RB).

#### Thaumatophyllum saxicola

Taxon classificationPlantaeAlismatalesAraceae

(Krause) Sakur., Calazans & Mayo comb. nov.

urn:lsid:ipni.org:names:77178489-1

Philodendron saxicola Krause, Pflanzenr. IV, 23Db: 133. 1913.
Type. Brazil, Bahia, Serra do Sincorá, Nov. 1906, *E. Ule 7568* (holotype: B; isotype L).

#### Thaumatophyllum solimoesense

Taxon classificationPlantaeAlismatalesAraceae

(A.C. Smith) Sakur., Calazans & Mayo comb. nov.

urn:lsid:ipni.org:names:77178490-1

Philodendron solimoesense A.C. Smith, J. Arnold Arbor. 20: 289. 1939.
Type. Brazil, Amazonas, São Paulo de Olivença, basin of Creek Belem, Oct-Dec. 1936, *B.A. Krukoff 8861* (holotype: NY; isotype: F).

#### Thaumatophyllum speciosum

Taxon classificationPlantaeAlismatalesAraceae

(Schott ex Endl.) Sakur., Calazans & Mayo comb. nov.

urn:lsid:ipni.org:names:77178501-1

Philodendron speciosum Schott ex Endl., Gen. Pl. 1(3): 237. 1837.
Type. illustration in Schott Icones Aroideae N° 2522 (lectotype, designated by Sakuragui et al. 2011); Brazil, Minas Gerais, Descoberto, 10 Nov. 2001, *V.R. Almeida 18* (epitype, designated by Sakuragui et al. 2011: CESJ, RB).

#### Thaumatophyllum spruceanum

Taxon classificationPlantaeAlismatalesAraceae

Schott, Bonplandia (Hannover) 7: 31. 1859.

Philodendron spruceanum (Schott) G.M. Barroso, Arch. Jard. Bot. Rio de Janeiro 17: 14. 1962, *nom. illeg.*
Type. based on Thaumatophyllum spruceanum Schott.
Philodendron goeldii G.M. Barroso, Arch. Jard. Bot. Rio de Janeiro 15: 95. 1957.
Type. Brazil, Manaus, Igarapé das Flores, 30 Sept. 1903, *A. Goeldi s.n.* (holotype: MG 3879).

##### Type.

Brazil, inundated forest in angle between Rio Negro and Solimões, 1851, *Spruce 120* (holotype: K).

#### Thaumatophyllum stenolobum

Taxon classificationPlantaeAlismatalesAraceae

(E.G. Gonç.) Sakur., Calazans & Mayo comb. nov.

urn:lsid:ipni.org:names:77178491-1

Philodendron stenolobum E.G. Gonç., Aroideana 25: 3. 2002 publ. 2003.
Type. Brazil, Espírito Santo, Colatina, road to São Domingos, 10 Oct. 2000, *E.G. Gonçalves et al. 567* (holotype: UB).

#### Thaumatophyllum tweedieanum

Taxon classificationPlantaeAlismatalesAraceae

(Schott) Sakur., Calazans & Mayo comb. nov.

urn:lsid:ipni.org:names:77178492-1

Philodendron tweedieanum Schott, Bonplandia (Hannover) 7: 29. 1859.
Type. Argentina, Entre Rios, delta region of Rio Paraná, *J. Tweedie s.n.* (**lectotype, designated here**: K; isolectotype: LE).
Philodendron dubium Chodat & Vischer, Bull. Soc. Bot. Genève 11: 295. 1919 publ. 1920.
Type. Paraguay, prope San Bernardino, *E. Hassler 1713* (**lectotype, designated here**: G); Paraguay, Lago Ypacaraí, *R.H. Chodat & W. Vischer 359* (remaining syntype: G, not found).

#### Thaumatophyllum uliginosum

Taxon classificationPlantaeAlismatalesAraceae

(Mayo) Sakur., Calazans & Mayo comb. nov.

urn:lsid:ipni.org:names:77178502-1

Philodendron uliginosum Mayo, Kew Bull. 46: 666. 1991.
Type. Brazil, Minas Gerais, Santana do Riacho, 25 Oct. 1974, *G. Hatschbach & Koszicki 35350* (holotype: MBM; isotypes K, US).

#### Thaumatophyllum undulatum

Taxon classificationPlantaeAlismatalesAraceae

(Engl.) Sakur., Calazans & Mayo comb. nov.

urn:lsid:ipni.org:names:77178493-1

Philodendron undulatum Engl., Monogr. Phan. 2: 428. 1879.
Type. Paraguay, Aregua plains, Jul. 1875, *B. Balansa 576* (**lectotype, designated here**: P; isolectotype: G, not found).
Philodendron eichleri Engl., Bot. Jahrb. Syst. 26: 556. 1899.
Type. Brazil, Minas Gerais, Carandaí, 15 Nov. 1887, *A.F.M. Glaziou 17332* (lectotype, designated by Sakuragui et al. 2011: K; remaining syntype: P).
Philodendron petraeum var. tobatiense Chodat & Vischer, Bull. Soc. Bot. Genève 11: 297. 1919 publ. 1920.
Type. Paraguay, Cerro Tobaty, *R.H. Chodat & W. Vischer 350* (holotype: G).

#### Thaumatophyllum venezuelense

Taxon classificationPlantaeAlismatalesAraceae

(Bunting) Sakur., Calazans & Mayo comb. nov.

urn:lsid:ipni.org:names:77178494-1

Philodendron venezuelense Bunting, Acta Bot. Venez. 10: 315. 1975.
Type. Venezuela, Territorio Federal Amazonas, Departamento Casiquiare, environs of Yavita on the Temi and near the Yavita-Pimichín road, 6-19 Jul. 1969, *Bunting et al. 3864* (holotype: MY; isotypes: NY, U).

#### Thaumatophyllum williamsii

Taxon classificationPlantaeAlismatalesAraceae

(J.D. Hooker) Sakur., Calazans & Mayo comb. nov.

urn:lsid:ipni.org:names:77178503-1

Philodendron williamsii J.D. Hooker, Bot. Mag. 97: t. 5899. 1871.
Type. Brazil, Bahia, region of Salvador, cultivated at Kew, Aug. 1870, *Williams s.n.* (holotype: K).

#### Thaumatophyllum xanadu

Taxon classificationPlantaeAlismatalesAraceae

(Croat, Mayo & J. Boos) Sakur., Calazans & Mayo comb. nov.

urn:lsid:ipni.org:names:77178495-1

Philodendron xanadu Croat, Mayo & J. Boos, Aroideana 25: 63. 2002 publ. 2003.
Type. origin unknown, based on plant cultivated in Wellington, West Palm Beach, Florida, *T.B. Croat 81537* (holotype: K; isotypes: B, F, COL, GH, INPA, K, MO, NY, R, RSA, SP, TRIN, UB, US).

### Key to the species of *Thaumatophyllum*

(adapted from Mayo (1991) and Gonçalves and Salviani (2002))

**Table:** 

1	Leaf blade transverse-cordiform in outline, pedately compound	**2**
–	Leaf blade cordiform-sagittate, sagittate or hastate in outline, margins entire, repand, sinuately lobed, pinnatifid or bipinnatifid	**3**
2	Ovary locules 3–4; leaflets 8–11, central leaflet 10–17 cm long; occurring on rocks in semi-arid areas or terrestrial in coastal restinga scrub on sand; usually in association with populations of Bromeliaceae; northeast Brazil	***T. leal-costae***
–	Ovary locules 10–26; leaflets 10–20, central leaflet 18–50 cm long; hemiepiphytic or terrestrial; most common along river margins; Amazon basin	***T. spruceanum***
3	Leaf margin sinuately lobed, pinnatifid or bipinnatifid	**4**
–	Leaf margin entire or repand or, if sinuately lobed, then peduncle 16 cm long or more	**11**
4	Leaf margin bipinnatifid, rarely pinnatifid but then with primary lateral veins of anterior division (5-)6–9(-10) per side; leaf blade over 50 cm long, primary lateral lobes (12-)17–35(-55) cm long	**5**
–	Leaf margin undulate or pinnatifid; if pinnatifid, then with primary lateral veins of anterior division 3–4(-10) per side; leaf blade up to 50 cm long, usually smaller; primary lateral lobes 5–17.5 cm long	**7**
5	Petioles flattened or slightly convex adaxially; intravaginal squamules never persistent, foliage leaf scars always concolorous with the internodes; uplands of Cerrado (Minas Gerais, Bahia and Goiás states – 700–1200 m)	***T. lundii***
–	Petioles conspicuously sulcate adaxially; intravaginal squamules persistent, very rarely deciduous (if deciduous, then foliage leaf scars discolorous with the internodes)	**6**
6	Intravaginal squamules numerous and dense, 5–12 mm long, 2–4 mm wide at base, persistent but easily detachable, rarely deciduous; female portion of the spadix adnate to the spathe for 60–80% of its length; southern and western (coastal) Brazil, Argentina, Paraguay	***T. bipinnatifidum***
–	Intravaginal squamules few and scattered, robust, 8–20 x 5–10 mm, always persistent, hardly detachable; female portion of the spadix adnate to spathe for 40–50% of its length; northern Goiás and possibly Mato Grosso states	***T. mello-barretoanum***
7	Plants aquatic or rarely terrestrial; leaf margin sinuately lobed (sinuses penetrating less than halfway to midrib), primary lateral lobes of anterior division 1.5–6.5(-14) cm long, usually oblique and turned towards leaf apex; female zone of the spadix (1.5-)4–5 cm long	**8**
–	Plant rupiculous or terrestrial; leaf margin pinnatifid (sinuses penetrating at least halfway to midrib), primary lateral lobes of anterior division 5–17.5 cm long, not oblique, female zone of the spadix 1.4–3.4 cm long	**9**
8	Stems with long and thorn-like intravaginal squamules; leaf blades never erect in living plants; Argentina, Paraguay, Bolivia and Brazil (South and South-eastern)	***T. undulatum***
–	Stems without persistent intravaginal squamules; leaves always erect or semi-erect in living plants; Eastern Brazil (northern Espírito Santo and southern Bahia)	***T. paludicola***
9	Petiole green or glaucous green at apex; leaf blade broadly ovate in outline, dark to subglossy green, sometimes glaucous, primary lateral lobes 3–4(-5); spathe 6.4–16 cm long, green outside, opened at anthesis; ovary locules 4–8(-11)	**10**
–	Petiole purplish at apex; leaf blade triangular to ovate in outline, glossy dark green, primary lateral lobes 5–10; spathe (8.2-)12–18 cm long, dark purple outside, tightly clasped around spadix at anthesis; ovary locules (6-)7–8	***T. xanadu***
10	Leaf blade (32-)35–50 cm long, primary lateral lobes of anterior division 3.5–6.3(-7.5) cm wide, distance between sinuses and midrib progressively greater towards base of anterior division; fertile male zone of the spadix 1.5–2.2 cm diam.	***T. saxicola***
–	Leaf blade 17-33 cm long, primary lateral lobes of anterior division 1.4–3.7(-7.4) cm wide, distance between sinuses and midrib usually becoming progressively less towards base of anterior division; fertile male zone of spadix 0.85–1.3 cm diam.	***T. adamantinum***
11	Overall length of adult leaf blade more than 60 cm (sometimes 50–60 cm in *T. solimoesense*); petiole apex often minutely rugose-verruculate (may be smooth in *T. stenolobum*)	**12**
–	Overall length of leaf blade less than 60 cm, petiole apex smooth, never occurring in Amazonia	**16**
12	Species from Eastern Brazil; stamens 6 mm long or more; staminodes more than 1.6 mm wide at apex, less than 2.5× longer than wide; ovary locules 6–13 per ovary	**13**
–	Species from Amazonia; stamens less than 6 mm long; staminodes less than 1.6 mm wide at apex, more than 2.5× longer than wide; ovary locules 17–34(-47) per ovary	**15**
13	Leaf blade narrowly sagittate, sometimes subhastate; anterior division 2.1–3.3× longer than wide; intravaginal squamules deciduous	***T. stenolobum***
–	Leaf blade broadly sagittate; anterior division 1–1.5× longer than wide; intravaginal squamules small but persistent	**14**
14	Leaf blade less than twice as long as wide; spathe outside lacking extrafloral nectaries, inside carmine magenta at anthesis; central style dome lacking	***T. speciosum***
–	Leaf blade more than twice as long as wide; spathe outside with punctate, pale brown extrafloral nectaries, inside cream-white at anthesis; central style dome present	***T. williamsii***
15	Cataphylls persistent; primary lateral veins of anterior division of leaf blade (5-)6–7; ovary locules 17–22; style elongated, distinctly narrower than ovary and lacking an axial canal	***T. venezuelense***
–	Cataphylls deciduous; primary lateral veins of anterior division of leaf blade (3-)4–5(-6); ovary locules 26–34(-47); style short, as broad as ovary with an axial canal or cavity which is very conspicuous in fruit	***T. solimoesense***
16	Peduncle subequal to twice as long as spathe; plants aquatic or rupiculous, aerial portion of the stem unbranched; internodes shorter than prophyll scars	**17**
–	Peduncle only about one third of spathe length; plant hemi-epiphytic or terrestrial; aerial stem branching frequently; internodes usually longer than prophyll scars	***T. corcovadense***
17	Leaf blade at least twice as long as broad; style longer than ovary	***T. dardanianum***
–	Leaf blade much less than twice as long as broad; style shorter than ovary	**18**
18	Intravaginal squamules abundant; broadly triangular, 3–12 mm long, (1.5-)3–7(-9) mm broad at base; style body as wide as ovary	***T. brasiliense***
–	Intravaginal squamules few, more narrowly triangular, 1.5–5 mm long, 0.5–2.5 mm broad at base; style body slightly narrower than ovary	**19**
19	Leaf blades subglossy to glaucous green, margins weakly repand; Argentina, Paraguay, Uruguay, South Brazil	**20**
–	Leaf blades dark glossy green, margins entire, rarely repand; central Brazil	***T. uliginosum***
20	Plants rupiculous, stem erect; prophyll deciduous; stylar central dome present	***T. petraeum***
–	Plants aquatic, stem decumbent or rhizomatous subterranean; prophyll marcescent and persistent; stylar central dome absent	***T. tweedieanum***

## Supplementary Material

XML Treatment for Thaumatophyllum

XML Treatment for Thaumatophyllum adamantinum

XML Treatment for Thaumatophyllum bipinnatifidum

XML Treatment for Thaumatophyllum brasiliense

XML Treatment for Thaumatophyllum corcovadense

XML Treatment for Thaumatophyllum dardanianum

XML Treatment for Thaumatophyllum leal-costae

XML Treatment for Thaumatophyllum lundii

XML Treatment for Thaumatophyllum mello-barretoanum

XML Treatment for Thaumatophyllum paludicola

XML Treatment for Thaumatophyllum petraeum

XML Treatment for Thaumatophyllum saxicola

XML Treatment for Thaumatophyllum solimoesense

XML Treatment for Thaumatophyllum speciosum

XML Treatment for Thaumatophyllum spruceanum

XML Treatment for Thaumatophyllum stenolobum

XML Treatment for Thaumatophyllum tweedieanum

XML Treatment for Thaumatophyllum uliginosum

XML Treatment for Thaumatophyllum undulatum

XML Treatment for Thaumatophyllum venezuelense

XML Treatment for Thaumatophyllum williamsii

XML Treatment for Thaumatophyllum xanadu

## Supplementary materials

Supplementary material 1 Taxon sampling, voucher information and GenBank Data type: molecular data Explanation note: Taxon sampling, voucher information and GenBank accession numbers of *Philodendron*, *Homalomena* and outgroup species. This is an open access article distributed under the terms of the Creative Commons Attribution License (CC BY 4.0), which permits unrestricted use, distribution, and reproduction in any medium, provided the original author and source are credited.Cassia Mônica Sakuragui, Luana Silva Braucks Calazans, Leticia Loss de Oliveira, Érica Barroso de Morais, Ana Maria Benko-Iseppon, Santelmo Vasconcelos, Carlos Eduardo Guerra Schrago, Simon Joseph Mayo
