# Supplementary material for: Recognition of the genus Thaumatophyllum Schott − formerly Philodendron subg. Meconostigma (Araceae) − based on molecular and morphological evidence
Source: PhytoKeys. 2018 May 2;(98):51–71. doi: 10.3897/phytokeys.98.25044 (PMC5943393; doi:10.3897/phytokeys.98.25044)
Supplement: Supplementary material 1 — Taxon sampling, voucher information and GenBank [file phytokeys-98-051-s001.doc]

**Supplementary Material**

**Table 1.** Taxon sampling, voucher information and GenBank accession numbers of *Philodendron*, *Homalomena* and outgroup species.

| **Taxon sampling** | **Voucher - Herbarium** | **5’ *mat*K** | **3’ *mat*K and *trn*K intron** | ***trn*L intron** | ***trn*L-*trn*F intergenic spacer** | **ETS + 18S** |
| --- | --- | --- | --- | --- | --- | --- |
| **Outgroup** |  |  |  |  |  |  |
| *Cercestis* *afzelii* Schott | Chase 11690 - RBG Kew | - | - | KU727619* | KU727678* | - |
| *Cercestis camerunensis* (Ntépé-Nyamè) Bogner | Salazar 6305 - RBG Kew | - | - | KU727597* | KU727677* | - |
| *Cercestis kawennianus* (Engl.) N.E. Br. | Salazar 6306 - RBG Kew | - | - | KU727616* | KU727675* | - |
| *Cercestis* sp. | Morais 57 | KU739335* | - | KU727617* | KU727676* | - |
| *Culcasia* *rotundifolia* Bogner | Morais 58 | - | - | KU727618* | KU727682* | - |
| *Dieffenbachia* *elegans* A.M.E. Jonker & Jonker | Calazans 88 - RFA | KU739301* | KU739349* | - | KU727681* | - |
| *Furtadoa* *mixta* (Ridl.) M. Hotta | Sakuragui 1402 - RBG Kew | KU739329* | - | KU727594* | KU727627* | - |
| *Heteropsis* *flexuosa* (Kunth) G.S. Bunting | Morais 81 - RFA | KU739303* | - | KU727527* | KU727579* | - |
| *Montrichardia* *arborescens* (L.) Schott | Oliveira 53 - RFA | KU739300* | - | KU727526* | KU727576* | - |
| *Nephthytis* *afzelii* Schott | Chase 10692 | KU739336* | KU739351* | KU727599* | KU727674* | - |
| *Nephthytis* *poissoni* (Engl.) N.E.Br. | Chase 14862 - RBG Kew | KU739334* | - | KU727622* | KU727680* | - |
| *Nephthytis* *swainei* Bogner | Chase 14863 - RBG Kew | - | - | KU727623* | KU727679* | - |
| *Urospatha* *sagittifolia* (Rudge) Schott | Oliveira 47 - RFA | - | KU739317* | - | KU727577* | - |
| ***Homalomena*** |  |  |  |  |  |  |
| *H*. *aromatica* (Spreng.) Schott | Sakuragui 1368 - RBG Kew | - | - | KU727624* | KU727669* | - |
| *H.* *cochinchinensis* Engl. | Calazans 36 et al. - RB; Sakuragui 1369 - RBG Kew | KF971331 | KF981856 | KU727591* | KU727657* | DQ870560.1 |
|  |  |  |  |  |  |  |
|  |  |  |  |  |  |  |
| *H. expedita* A.Hay & Hersc. | - | JX024965.1 | JX024965.1 | - | KU727631* | - |
| *H. griffithii* (Schott) Hook. f. | - | KU739327* | - | - | - | - |
| *H. humilis* (Jack) Hook. f. | Sakuragui 1371 - RBG Kew | KU739328* | - | KU727592* | KU727659* | - |
| *H. magna* A. Hay | Chase 10691 - RBG Kew | AM920596.1 | AM920596.1 | KU727614* | KU727683* | - |
|  |  |  |  |  |  |  |
| *H. pendula* (Blume) Bakh. f. | Sakuragui 1372 - RBG Kew | - | - | KU727593* | KU727660* | - |
| *H. philippinensis* Engl. | - | - | - | - | - | DQ870564.1 |
|  |  |  |  |  |  |  |
| *H. rubescens* (Roxb.) Kunth |  | KU739290* | - | KU727520* | KU727547* | DQ870566.1 |
| *H. tenuispadix* Engl. | Sakuragui 1404 - RBG Kew | KU739326* | - | KU727598* | KU727653* | - |
| *H. wallichii* Schott | Sakuragui 1405 - RBG Kew | KU739324* | - | KU727625* | KU727645* | - |
|  |  |  |  |  |  |  |
| ***Adelonema*** |  |  |  |  |  |  |
| (*H. crinipes* Engl.) = *Adelonema crinipes* (Engl.) S. Y. Wong & Croat, |  | - | - | - | - | DQ870561.1 |
| *Adelonema erythropus* Schott | - | - | - | - | - | DQ870562.1 |
| *Adelonema panamensis Croat & Mansell* | - | - | - | - | - | DQ870563.1 |
| *Adelonema picturata* (Linden & André) S. Y. Wong & Croat =(*H. picturata* (Linden & André) Regel) | - | - | - | - | - | DQ870565.1 |
| *Adelonema wendlandii* (Schott) S. Y. Wong & Croat =(*H. wendlandii* Schott) | - | KU739283* | - | KU727528* | KU727543* | DQ870567.1 |
| ***P*. subg. *Meconostigma*** |  |  |  |  |  |  |
| *P. adamantinum* Mart. ex Schott | Bastos 20 et al., Calazans 40 - RFA | KU739331* | - | KU727607* | KU727629* | KF895425 |
| *P. bipinnatifidum* Schott ex Endl. | Calazans 4 - RB | KF971323 | KF981849 | KU727609* | KU727567* | KF895410 |
| *P. brasiliense* Engl. | Bastos 21 et al., Calazans 23 - RFA | KU739325* | - | KU727513* | KU727551* | KF895413 |
| *P. corcovadense* Kunth | Calazans 17 et al., Morais 23 - RFA | KF971324 | KF981850 | KU727515* | KU727568* | KF895417 |
| *P. dardanianum* Mayo | Calazans 22 et al. - RB | - | - | KU727587* | KU727661* | KF895411 |
| *P. goeldii* G.M. Barroso | Calazans 72 et al. - RB | - | - | - | KU727662* | KF895428 |
| *P. leal-costae* Mayo & G.M. Barroso | Calazans 47 - HUEFS | - | - | - | KU727630* | KF895427.1 |
| *P. lundii* Warm. | Calazans 41 et al. - RB | KF971332 | KF971332 | KU727512* | KU727546* | KF895420.1 |
| *P. mello-barretoanum* R. Burle-Marx ex G.M. Barroso | Morais 51 - RB | - | KU739347* | KU727588* | KU727655* | KF895423 |
| *P. paludicola* E.G. Gonç. & Salviani | Calazans 38 et al. - RB | - | - | KU727612* | KU727654* | KF895412 |
| *P. petraeum* Chodat & Vischer | Calazans 28 et al. - RB | KF981853 | KF981853 | - | KU727667* | KF895422 |
| *P. saxicola* K. Krause | Calazans 50 & Morais - RFA | KF971327 | KF981854 | KU727521* | KU727545* | KF895426 |
| *P. solimoesense* A.C. Sm. | Oliveira et al. 57 - INPA | KF971333 | KF971333 | KU727621* | KU727668* | KF895429 |
| *P. speciosum* Schott ex Endl. | Morais 38 - RFA | - | - | KU727590* | KU727684* | KF895414 |
| *P. stenolobum* E.G. Gonç. | Bastos 22 et al. - RFA | KF971334 | KF971334 | KU727608* | KU727664* | KF895424 |
| *P. tweedieanum* Schott | Calazans 37 et al. - RB | - | - | KU727611* | KU727656* | KF895421 |
| *P. uliginosum* Mayo | Calazans 19 et al. - RB | - | - | - | KU727651* | KF895419 |
| *P. undulatum* Engl. | Calazans 7 - RB | KF971328 | KF981855 | KU727508* | KU727663* | KF895418 |
| *P. venezuelense* G.S. Bunting | Calazans 26 - RB | KF971329 | KF971329 | KU727610* | KU727665* | KF895415 |
| *P. williamsii* Hook. f. | Calazans 54 & Morais - HUEFS | KF971330 | KF971330 | KU727589* | KU727666* | KF895416 |
| *P. xanadu* Croat, Mayo & J. Boos | L. Mayano s/n | - | - | KU727613* | KU727649* | KF895409 |
| ***P*. subg. *Philodendron*** |  |  |  |  |  |  |
| *P. acutatum* Schott | Bastos 14, Calazans 10 - RB | KU739289* | KU739342* | KU727510* | KU727549* | DQ870570.1 |
| *P. aemulum* Schott | Sakuragui 629 - RBG Kew | KU739338* | - | - | KU727641* | - |
| *P. angustilobum* Croat & Grayum | Sakuragui 641 - RBG Kew | - | - | KU727583* | KU727634* | - |
| *P. angustisectum* Engl. | - | - | - | - | - | DQ870576.1 |
| *P. annulatum* Croat | Sakuragui 1378 - RBG Kew | KU739337* | KU739350* | KU727580* | KU727647* | - |
| *P. appendiculatum* Nadruz & Mayo | Calazans 105, Calazans 133 - RB | KU739332* | KU739313* | KU727503* | KU727539* | - |
| *P. asplundii* Croat & M.L. Soares | Morais 91 - RB | KU739296* | - | KU727497* | KU727537* | - |
| *P. auriculatum* Standl. & L.O. Williams | Sakuragui 1379 - RBG Kew | KU739322* | - | KU727595* | KU727648* | - |
| *P. barrosoanum* G.S. Bunting | Oliveira 41 - RB | KU739282* | - | - | KU727558* | DQ870577.1 |
| *P. billietiae* Croat | Oliveira 38, Oliveira 40 - RB | KU739298* | KU739309* | KU727509* | KU727531* | DQ870578.1 |
| *P. brevispathum* Schott | - | - | - | - | - | DQ870579.1 |
| *P. burlemarxii* G.M. Barroso | Bastos 5 - RB | - | KU739306* | KU727499* | KU727560* | - |
| *P. callosum* K. Krause | Bastos 12 - RB; Sakuragui 632 - RBG Kew | KU739285* | - | KU727518* | KU727673* | DQ870580.1 |
| *P. camposportanum* G.M. Barroso | Bastos 23 - RB | - | - | - | KU727571* | - |
| *P. cannifolium* (Dryand. ex Sims) Sweet | - | - | - | - | - | DQ870581.1 |
| *P. cordatum* Kunth | Calazans 59, Wängler 268 | KU739277* | KU739304* | KU727487* | KU727540* | - |
| *P. crassinervium* Lindl. | Calazans 13 - RB; Sakuragui 1383 - RBG Kew | KU739330* | KU739346* | KU727596* | KU727670* | DQ870582.1 |
| *P. davidsonii* Croat | - | - | - | - | - | DQ870583.1 |
| *P. deltoideum* Poepp. | Chase 10891 - RBG Kew | AM920597.1 | AM920597.1 | KU727585* | KU727646* | - |
| *P. distantilobum* K. Krause | - | - | - | - | - | DQ870584.1 |
| *P. edmundoi* G.M. Barroso | Calazans 30, Calazans 43 - RB | KU739295* | - | - | KU727570* | - |
| *P. elaphoglossoides* Schott | Morais 105 - RB | KU739294* | - | KU727507* | - | - |
| *P. erubescens* K. Koch & Augustin | Pellegrini 10 - RB | KU739268* | - | KU727514* | KU727554* | DQ870585.1 |
| *P. eximium* Schott | Pellegrini 109, Calazans 126 - RB | KU739318* | KU739315* | KU727489* | KU727555* | - |
| *P. findens* Croat & Grayum | - | - | - | - | - | DQ870586.1 |
| *P. fragrantissimum* (Hook.) G. Don | Morais 64 - RB; Sakuragui 1385 - RBG Kew | JQ586639.1 | JQ586639.1 | KU727504* | KU727544* | DQ870587.1 |
| *P. glaziovii* Hook. f. | Sakuragui 639 - RBG Kew | - | KU739352* | KU727605* | KU727632* | DQ870588.1 |
| *P. gloriosum* André | - | KU739273* | - | KU727498* | KU727564* | DQ870589.1 |
| *P. grandifolium* (Jacq.) Schott | Bastos 16 - RB | - | - | KU727490* | KU727561* | DQ870590.1 |
| *P. grandipes* K. Krause | - | - | - | - | - | DQ870591.1 |
| *P. hastatum* K. Koch & Sellow | Pellegrini 57 - RB | KU739279* | - | KU727492* | KU727538* | KU727767 |
| *P. hederaceum* (Jacq.) Schott | Calazans 154 - RB | DQ401355.1 | DQ401355.1 | KU727606* | KU727672* | DQ870613.1 |
| *P. heleniae* Croat | - | - | - | - | - | DQ870592.1 |
| *P. hopkinsianum* M.L. Soares & Mayo | Oliveira 34, Morais 106 - RB | KU739291* | - | KU727506* | KU727542* | - |
| *P. hylaeae* G.S. Bunting | Oliveira 30 - RB | - | - | - | KU727552* | DQ870593.1 |
| *P. imbe* Schott ex Endl. | - | KU739299* | - | - | KU727534* | DQ870595.1 |
| *P. inconcinnum* Schott | Sakuragui 636 - RBG Kew | - | - | KU727584* | KU727633* | - |
| *P. insigne* Schott | Calazans 29 - RB; Sakuragui 1389 - RBG Kew | KU739286* | - | KU727517* | KU727572* | DQ870596.1 |
| *P. krugii* Engl. | Sakuragui 638 - RBG Kew | - | - | KU727602* | KU727638* | - |
| *P. lazorii* Croat | - | - | - | - | - | DQ870597.1 |
| *P. lindenii* Schott | - | - | - | - | - | DQ870598.1 |
| *P. loefgrenii* Engl. | Bastos 28 - RB | - | - | - | - | DQ870600.1 |
| *P. longilaminatum* Schott | Calazans 25 - RB; Sakuragui 1392 - RBG Kew | - | KU739339* | KU727601* | KU727671* | - |
| *P. longistilum* K. Krause | Bastos 10 - RB | KU739276* | - | KU727491* | - | DQ870601.1 |
| *P. malesevichiae* Croat | - | - | - | - | - | DQ870602.1 |
| *P. martianum* Engl. | Calazans 32 - RB | - | KU739311* | KU727488* | KU727559* | - |
| *P. maximum* K. Krause | Calazans 77 - RB | - | KU739307* | KU727524* | KU727557* | - |
| *P. megalophyllum* Schott | Oliveira 44, Morais 89 - RB | KU739274* | KU739310* | KU727494* | KU727566* | - |
| *P. melanochrysum* Linden & André | Sakuragui 1393 - RBG Kew | KU739323* | KU739341* | - | KU727652* | - |
| *P. melinonii* Brongn. ex Regel | Calazans 33, Morais 88 - RB | KU739280* | - | - | KU727533* | DQ870603.1 |
| *P. micranthum* Poepp. ex Schott | Bastos 27 - RB | KU739281* | - | KU727495* | KU727535* | - |
| *P. myrmecophyllum* Engl. | Sakuragui 640 - RBG Kew | - | - | KU727586* | KU727639* | - |
| *P. nadruzianum* Sakur. | Calazans 58, Calazans 62 - RB | KU739271* | KU739308* | KU727500* | KU727565* | - |
| *P. ornatum* Schott | Oliveira 51, Calazans 103 - RB | - | - | - | KU727553* | DQ870604.1 |
| *P. pachyphyllum* K. Krause | Calazans 53 - RB | - | KU739343* | KU727604* | KU727640* | - |
| *P. panamense* K. Krause | - | - | - | - | - | DQ870605.1 |
| *P. panduriforme* (Kunth) Kunth | Morais 69 - RB | KU739297* | - | KU727525* | KU727573* | DQ870606.1 |
| *P. pedatum* (Hook.) Kunth | Calazans 48, Morais 82 - RB | KF971326 | KF981852 | KU727501* | KU727530* | DQ870607.1 |
| *P. pinnatifidum* (Willd.) Schott | - | - | - | - | - | DQ870608.1 |
| *P. pulchrum* G.M. Barroso | Morais 93 - RB | KU739275* | - | KU727502* | KU727536* | - |
| *P. quinquelobum* K. Krause | Morais 83, Calazans 74 - RFA | KU739272* | - | KU727529* | KU727578* | - |
| *P. radiatum* Schott | - | JX024994.1 | JX024994.1 | KU727582* | KU727637* | DQ870610.1 |
| *P. recurvifolium* Schott | Sakuragui 1003 - RBG Kew | - | - | KU727600* | KU727643* | - |
| *P. renauxii* Reitz | - | KU739278* | - | KU727493* | KU727541* | - |
| *P. rhizomatosum* Sakur. & Mayo | Sakuragui 637 - RBG Kew | KU739333* | - | - | KU727650* | - |
| *P. roseopetiolatum* Nadruz & Mayo | Calazans 106 - RFA | - | - | - | KU727562* | - |
| *P. ruizii* Schott | - | - | - | - | - | DQ870611.1 |
| *P. ruthianum* Nadruz | Antas 10 - RB | - | KU739345* | - | - | - |
| *P. sagittifolium* Liebm. | - | - | - | - | - | DQ870612.1 |
| *P. simmondsii* Mayo | Sakuragui 642 - RBG Kew | KU739320* | KU739340* | KU727581* | KU727636* | - |
| *P. smithii* Engl. | - | - | - | - | - | DQ870616.1 |
| *P. sphalerum* Schott | Calazans 76, Calazans 95 - RFA | KU739269* | - | KU727511* | KU727532* | - |
| *P. squamiferum* Poepp. | Morais 63 - RFA; Sakuragui 1400 - RBG Kew | KU739288* | KU739344* | KU727516* | KU727548* | DQ870617.1 |
| *P. stenophyllum* K. Krause | - | - | - | - | - | DQ870618.1 |
| *P. tortum* M.L. Soares & Mayo | Oliveira 36 - RFA | - | - | - | KU727556* | - |
| *P. toshibai* M.L. Soares & Mayo | Oliveira 49 - RFA | KU739270* | - | KU727496* | KU727563* | - |
| *P. tripartitum* (Jacq.) Schott | Chase 10894 - RBG Kew | KU739321* | KU739348* | KU727603* | KU727635* | DQ870620.1 |
| *P. verrucosum* L. Mathieu ex Schott | - | - | - | - | - | DQ870621.1 |
| *P. wendlandii* Schott | - | - | - | - | - | DQ870622.1 |
| *P. wittianum* Engl. | Oliveira 32 - RB | KU739292* | - | KU727505* | KU727550* | - |
| *P. wurdackii* G.S. Bunting | Morais 54 - RFA | KU739267* | - | KU727522* | - | - |
| *P. linnaei*Kunth, Enum | Calazans 27, Bastos 17 - RB | KU739287* | KU739314* | KU727519* | KU727569* | DQ870599.1 |
| ***P*. subg. *Pteromischum*** |  |  |  |  |  |  |
| *P. oblongum* (Vell.) Kunth | Morais 48, Calazans 127 - RFA | KF971325 | KF981851 | KU727523* | - | - |
| *P. ochrostemon* Schott | Calazans 119 - RFA | - | - | - | KU727575* | - |
| *P. placidum* Schott |  | - | - | - | - | DQ870609.1 |
| *P. propinquum* Schott | Calazans 122 - RFA | KU739284* | - | - | KU727574* | - |
| *P. rudgeanum* Schott | Calazans 156, Calazans 157 - RFA | - | - | KU727620* | KU727642* | DQ870568.1 |
| *P. schottii* subsp. *talamancae* (Engl.) Grayum | - | - | - | - | - | DQ870619.1 |
| *P. surinamense* (Miq.) Engl. | Sakuragui 1001 - RBG Kew | - | - | - | KU727628* | - |

Figure 1: Gene trees estimated in Mr. Bayes. The posterior probabilities are indicated in the nodes. A. ETS and 18S gene tree; B. *mat*K 5’ portion gene tree; C. *mat*K 3’ portion and *trn*K gene tree; D. *trn*L intron gene tree; E. *trn*L-*trn*F intergenic spacer gene tree.
